# Novel hot spring Thermoproteota support vertical inheritance of ammonia oxidation and carbon fixation in Nitrososphaeria

**DOI:** 10.1099/acmi.0.000931.v4

**Published:** 2025-04-29

**Authors:** T. Slosser, M. Wenick, E. Markert, E. Trembath-Reichert, L. M. Ward

**Affiliations:** 1 Department of Geosciences, Smith College, Northampton, MA, USA; 2 School of Earth and Space Exploration, Arizona State University, Tempe, AZ, USA

**Keywords:** 3HP/4HB, ammonia monooxygenase, nitrification, phylogenomics

## Abstract

Aerobic ammonia oxidation is crucial to the nitrogen cycle and is only known to be performed by a small number of bacterial lineages [ammonia-oxidizing bacteria (AOB)] and a single lineage of archaea belonging to the *Nitrososphaeria* class of *Thermoproteota* [ammonia-oxidizing Archaea (AOA)]. Most cultivated AOA originate from marine or soil environments, but this may capture only a limited subset of the full diversity of this clade. Here, we describe several genomes of AOA from metagenomic sequencing of a hot spring microbial mat, representing several poorly characterized basal lineages that may be important for understanding the early evolution of archaeal ammonia oxidation. These genomes include a novel genus most closely related to *Nitrososphaera* as well as novel species belonging to the genera *Nitrosotenuis*, *Nitrososphaera* and *Nitrosotalea*. Furthermore, the distributions and phylogenetic relationships of key metabolic genes support a history of vertical inheritance of ammonia oxidation and carbon fixation from the last common ancestor of crown group AOA.

## Data Summary

All metagenomic sequence data are available via the JGI Genome Portal (https://genome.jgi.doe.gov/portal/Metchahotsprings/Metchahotsprings.info.html). Raw sequence data and draft genomes are available through NCBI under project ID PRJNA1090451. Raw sequence data are listed in the SRA database under accession numbers SRX26069680–SRX26069696. Metagenome-assembled genomes are listed in the WGS database under accessions JBDUUJ–JDPUUP. Supplemental information is available via the Microbiology Society’s Figshare data repository under manuscript number https://doi.org/10.6084/m9.figshare.27204708.v1[1]

## Introduction

The biogeochemical nitrogen cycle is essential both to convert relatively inert atmospheric N_2_ into bioavailable forms and to return fixed nitrogen to the atmosphere to maintain long-term steady state (e.g. [2]). Ammonia oxidation is only known to occur in four extant clades, which include a single lineage of archaea [ammonia-oxidizing archaea (AOA)] and three known lineages of ammonia-oxidizing bacteria (AOB). In the most current version of the Genome Taxonomy Database (GTDB; the taxonomic system that we use throughout this work), AOA are restricted to a clade within the *Nitrososphaerales* order of phylum *Thaumarchaeota* [3]. AOB include two distinct lineages of ammonia-oxidizing *Pseudomonadota* (formerly *Proteobacteria*) and one clade of commamox (complete ammonia oxidation to nitrate) bacteria in the *Nitrospira* class of *Nitrospirota* [4]. Though AOB and AOA both perform the first step of ammonia oxidation using the ammonia monooxygenase (AMO) enzyme, divergences in AMO structure and in the downstream biochemical pathways employed by these groups affect the environmental signals and distributions of different ammonia oxidizers. In particular, variations in oxygen tolerance, carbon fixation efficiency and substrate affinity [5,10] carry strong significance for the ecological niches [11,12] and primary productivity of AOB and AOA [4,13, 14].

Of particular note to this study, the discoveries of oligotrophic and thermophilic members of the AOA within the last two decades have expanded the potential range of ammonia oxidation into habitats where AOB have not been detected, including high-temperature environments like hot springs (e.g. [15,18]). Although the presence and significance of AOA in terrestrial hot springs are now widely recognized (e.g. [11,19]), only a few thermophilic AOA species have been previously characterized in detail, potentially leaving large gaps in our understanding of the clade’s taxonomic and metabolic diversity [15,20]. A thorough genome-resolved metagenomic sampling of geographically and geochemically diverse hot springs is essential for understanding the diversity, phylogenetic distribution and evolutionary history of microbial metabolic pathways (e.g. [21]). This issue is further exacerbated by biases in hot spring research in general, which has disproportionately drawn from well-studied springs in Yellowstone National Park and their associated geochemical conditions (e.g. [22,23]) leading to a relative dearth of knowledge about hot springs elsewhere in the world. Recent years have seen an increase in sampling in some regions (e.g. [24]), yet the southern hemisphere remains underrepresented in available datasets.

Here, we describe several metagenome-assembled genomes (MAGs) of novel AOA from hot spring microbial mats in Aotearoa New Zealand, substantially improving genomic sampling across basal AOA clades. Organismic and metabolic protein phylogenies incorporating these MAGs provide further support to the hypothesis that ammonia oxidation and carbon fixation via the 3HP/4HB pathway have been vertically inherited from the last common ancestor of extant AOA.

## Methods

Sample collection, processing and sequencing were performed as described previously [25] and below. Sixteen samples were collected from Waikite Valley hot spring in the Taupo Volcanic Zone of Aotearoa New Zealand in March 2018. Small (~0.25 cm^3^) samples of microbial mat were collected in duplicate from eight points along a transect along the flow path of the hot spring, starting near the source and proceeding downstream where the spring water was progressively cooler and more oxidized. All samples were derived from dense, leathery yellow- or green-coloured microbial mats submerged in pH 8 geothermal water along the outflow of the spring with temperatures ranging from 37.0 to 61.8 °C (additional geochemical parameters available through the 1000 Springs Project [26]). Site characterization, sample collection and processing followed methods described previously [21,27] and summarized here. Samples were immediately lysed, and DNA was preserved in the field using a Zymo Terralyzer BashingBead Matrix and Xpedition Lysis Buffer [28,29]. Cells were disrupted immediately following sampling by attaching sealed tubes to the blade of a cordless reciprocating saw and operating for 1 min. Following the return to the lab, microbial DNA was extracted and purified using a Zymo Biomics DNA Miniprep Kit following the manufacturer’s instructions. Library preparation and shotgun metagenomic sequencing were performed by the Joint Genome Institute via 2×151 bp Illumina NovaSeq. Raw sequence reads from each metagenomic sample were co-assembled with MegaHit v. 1.02 [30], and genome bins were constructed based on differential coverage using MetaBAT v2.15 [31] using the ‘meta-large’ presets. Annotation and basic analysis of MAGs was performed with RAST [32]. Taxonomic affinity of genome bins was determined using GTDB-Tk v2.4.0 [33], completeness and contamination were determined using CheckM v1.2.2 [34] and the presence of metabolic pathways of interest was predicted with MetaPOAP v1.0 [35]. Protein sequences of interest were targeted using the *tblastn* function of blast+ v0.2.14.1 [36] using reference sequences from the NCBI Protein database and a starting e value cutoff of 1e-20, aligned with muscle v5.1 [37] and manually trimmed in Jalview v2.11.4.1 [38] to remove portions of the alignment with low quality scores. Phylogenetic trees were calculated using RAxML v8.2.12 [39] on the Cipres science gateway [40], with transfer bootstrap support values calculated by BOOSTER v0.1.9 [41]. Trees were visualized using the Interactive Tree of Life viewer v7 [42]. All software was run using default parameters unless otherwise noted. Horizontal versus vertical inheritance of metabolic genes was inferred by comparison of organismal and metabolic protein phylogenies [43,45].

## Results

**Fig. 1.**
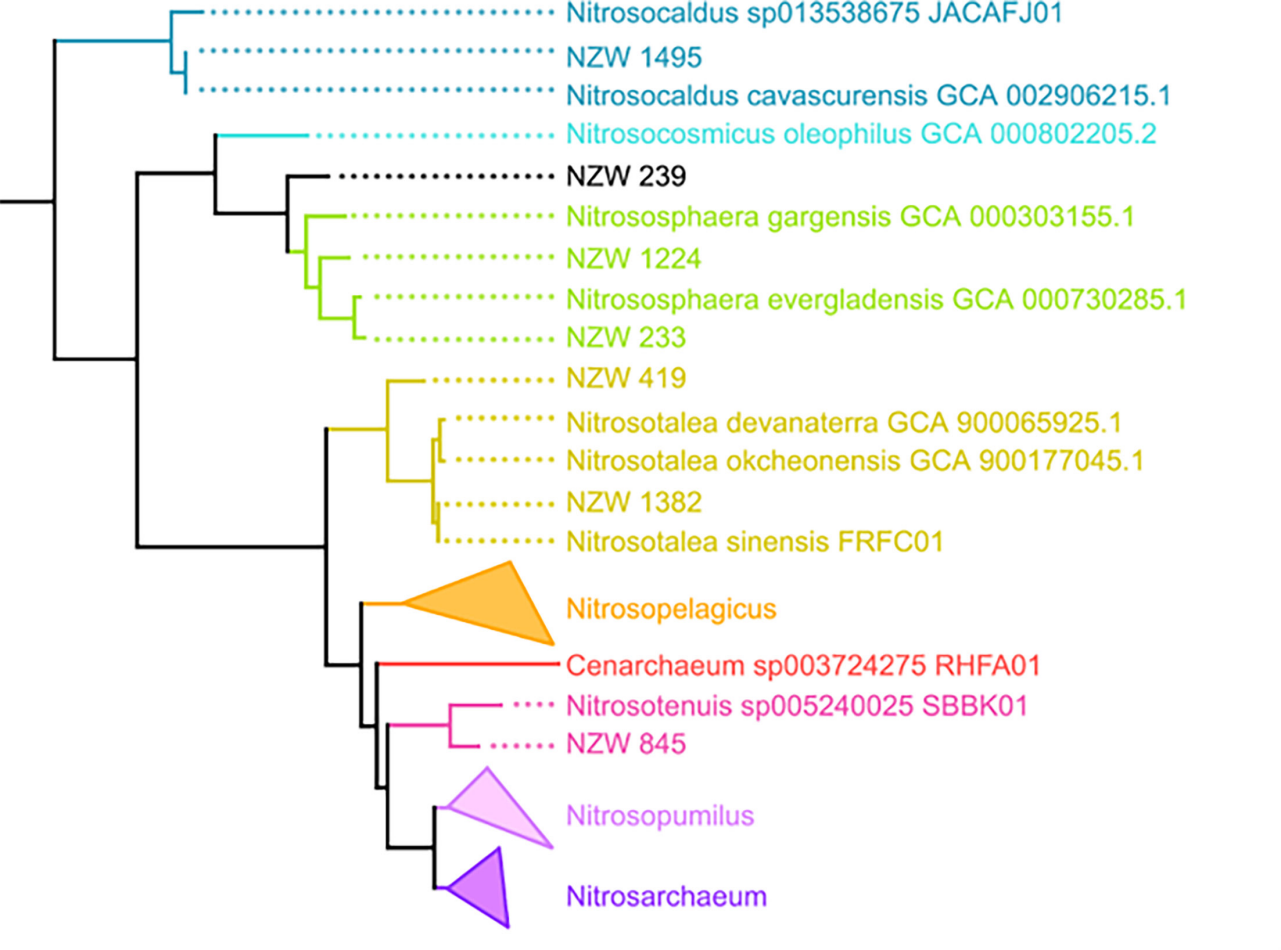
Organismal tree of MAGs described above along with well-characterized reference genomes, built with concatenated ribosomal protein sequences and rooted with outgroups from other archaeal phyla (*Micrarchaeota* and *Aenigmatarchaeota*). Colour coding is intended to highlight genus boundaries. The Terrestrial group include *Nitrosocaldus*, *Nitrososphaera*, *Nitrosotalea* and *Nitrosotenuis*, and the Shallow water group includes *Notrosopelagicus*, *Nitrosopumilus*, *Nitrosomarinus* and *Nitrosoarchaeum* [10]. Transfer bootstrap support for all nodes is greater than 0.75, and support is greater than 0.84 for all nodes representing divergence at the genus or higher taxonomic level.

Metagenomic sequencing of 16 samples along the transect of the Waikite Hot Spring produced ~686 gigabases of sequence. These were co-assembled into 10,193,979,067 bp across 9,997,303 contigs, with an N50 value of 2,370 bp. From this co-assembly, differential coverage-based binning produced seven MAGs predicted to belong to the *Nitrososphaeria* class of high or medium quality according to current standards [46], with most having completeness estimated to be over 97% and contamination under 2% according to CheckM (Table 1). These MAGs were assigned by GTDB-Tk to the *Nitrososphaerales* order, including members of previously characterized species (e.g. NZW_1495 as *Nitrosocaldus cavascurensis*) as well as some predicted to represent novel species (e.g. NZW_1224, *Nitrososphaera* sp.) or even genera (e.g. NZW_239, uncharacterized *Nitrososphaeraceae*; Fig. 1, Table 1). Reads mapping to these MAGs appeared in samples collected from 50.0 to 61.8 °C [25].

**Table 1.** List of MAGs and genome statistics

MAG ID	GTDB taxonomy	Complete-ness	Contami-nation	Strainheterogeneity	Genome size	No. of contigs	No. of proteins	GC	N50	Largest contig
NZW_1495	d__*Archaea* p__*Thermoproteota* c__*Nitrososphaeria* o__*Nitrososphaerales* f__*Nitrosocaldaceae* g__*Nitrosocaldus* s__*Nitrosocaldus*_*cavascurensis*	99.03	0	0	1,621,653	27	1,769	0.42	240,889	379,043
NZW_419	d__*Archaea* p__*Thermoproteota* c__*Nitrososphaeria* o__*Nitrososphaerales* f__*Nitrosopumilaceae* g__*Nitrosotalea* s__	99.51	14.89	15	1,764,298	80	2,171	0.36	79,256	362,437
NZW_1382	d__*Archaea* p__*Thermoproteota* c__*Nitrososphaeria* o__*Nitrososphaerales* f__*Nitrosopumilaceae* g__*Nitrosotalea* s__*Nitrosotalea*_*sinensis*	99.51	0.97	0	1,684,605	20	1,999	0.37	116,720	229,139
NZW_845	d__*Archaea* p__*Thermoproteota* c__*Nitrososphaeria* o__*Nitrososphaerales* f__*Nitrosopumilaceae* g__*Nitrosotenuis* s__	99.03	0.97	0	1,648,276	116	2,021	0.42	22,463	74,665
NZW_239	d__*Archaea* p__*Thermoproteota* c__*Nitrososphaeria* o__*Nitrososphaerales* f__*Nitrososphaeraceae* g__ s__	99.03	1.94	0	2,556,235	24	2,966	0.52	316,821	436,782
NZW_1224	d__*Archaea* p__*Thermoproteota* c__*Nitrososphaeria* o__*Nitrososphaerales* f__*Nitrososphaeraceae* g__*Nitrososphaera* s__	100	1.94	50	1,988,989	52	2,891	0.52	63,028	118,409
NZW_233	d__*Archaea* p__*Thermoproteota* c__*Nitrososphaeria* o__*Nitrososphaerales* f__*Nitrososphaeraceae* g__*Nitrososphaera* s__	76.74	15.53	90.48	1,737,296	274	2,284	0.55	7,506	24,244

**Fig. 2.**
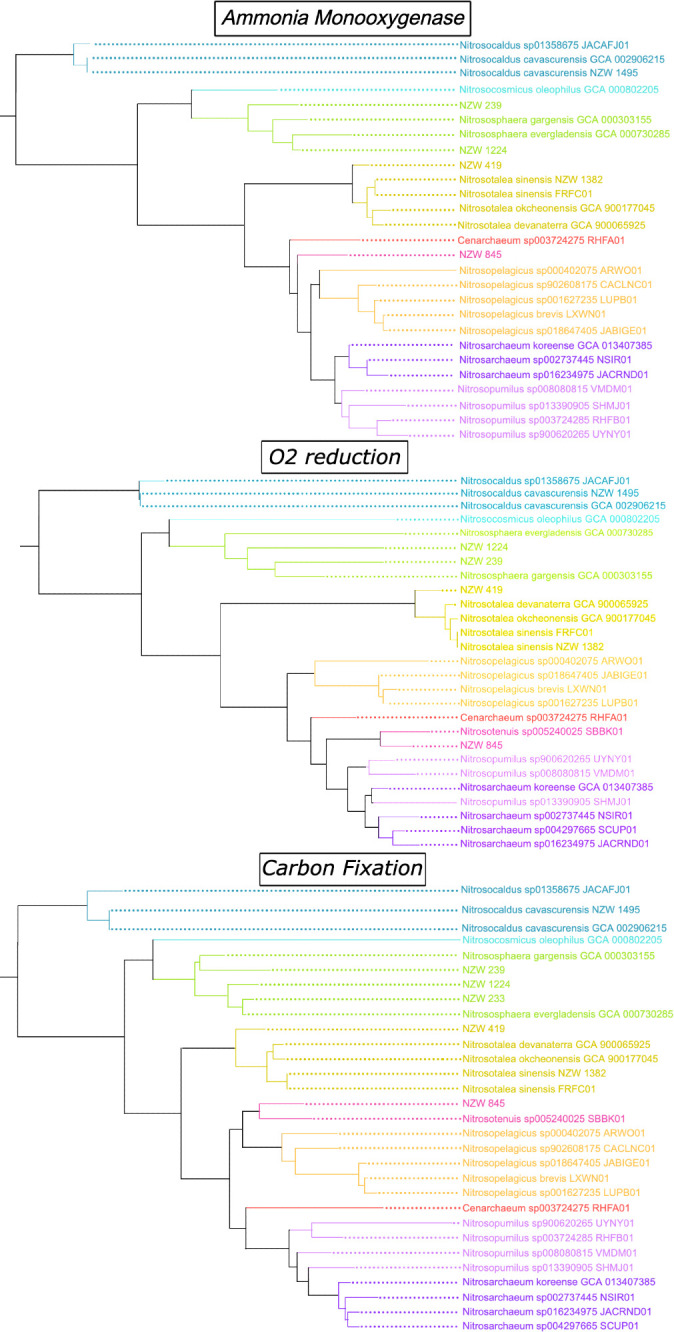
Protein phylogenies for AMO (concatenated AmoA, AmoB and AmoC), O_2_ reduction (subunit I of the A-family heme-copper oxidoreductase) and carbon fixation (concatenated Mut, Acc and Abf) from *Nitrososphaerales*, rooted with *Nitrosocaldus* as the outgroup. Colour code is retained from Fig. 1 to highlight (in)congruence of phylogenies.

The resultant MAGs contain genes encoding aerobic respiration via A-family heme-copper oxidoreductases, ammonia oxidation (as indicated by the well-characterized marker genes *amoABC* found in all known ammonia oxidizers [4]) and the 3HP/4HB pathway for carbon fixation (as indicated by marker genes including *mut*, *acc* and *abf*) (Fig. 2). The one exception is NZW_233, which is anomalously missing pathways for aerobic respiration and ammonia oxidation. It is worth noting that this sample had a contamination level of 15.53%, with over 90% of that predicted to be due to strain heterogeneity, suggesting that true contamination from distantly related organisms makes up less than 2% of the genome. The MetaPOAP False Negative for these pathways in NZW_233 is significantly low to support interpretations of secondary loss in this lineage (probability <0.015); however, the relatively low completeness of this MAG, the potential colocalization of genes involved in these pathways and the fact that the MAG is constructed almost entirely of small contigs (N50 <7,500, largest contig <25 k) may have led to higher odds of binning errors with this genome. Despite these caveats in its quality and analysis, NZW_233 was deemed important to include in this study as it is the first draft genome recovered of a lineage that appears to rank as a novel species within *Nitrososphaera* (Table 1). Moreover, very strong bootstrap support (1.0) in all phylogenies in which it appears (Data S1 and S4, Figs S1 and S4, available in the online Supplementary Material) further supports interpretations of no significant contamination in the sequences considered here.

## Discussion and conclusion

The evolutionary history of AOA has been confounded by a limited understanding of *Thermoproteota* diversity, with uneven sampling and cultivation leading to discrepancies between which AOA are most well-characterized and which are most ecologically significant [47]. Most of the diversity of previously characterized AOA is from the so-called ‘Shallow-water group’, (which includes *Nitrosopumilus*), with relatively few isolates or genomes representing the ‘Terrestrial group’, which appears to be a paraphyletic grade at the base of the AOA clade [10]; the phylogenetic placement of the ‘Terrestrial group’ suggests that they may be more representative of the most evolutionarily ancient AOA, with marine groups representing a much younger, evolutionarily derived radiation (e.g. [48]). Recent phylogenetic analyses also tend to infer a thermophilic ancestor for all AOA due to the basal placement of AOA from specifically geothermal environments (especially the lineage including *Nitrosocaldus* [10,49]). The added diversity of the genomes described here improves our sampling across the AOA evolutionary tree, both supporting the basal placement of *Nitrosocaldus* and adding new information about early-diverging ‘Terrestrial group’ lineages more generally. Our protein phylogenies further target specific biochemical mechanisms and demonstrate that the evolutionary relationships among proteins involved in respiration, ammonia oxidation and carbon fixation are broadly congruent with organismal relationships (Figs1  2, Data S2–S4 and Figs S2–S4), which suggests that these traits were vertically inherited rather than acquired via horizontal gene transfer. Thus, expanded hot spring sampling of *Nitrososphaerales* supports vertical inheritance from an aerobic, ammonia-oxidizing last common ancestor capable of carbon fixation.

These early divergences are key to our understanding of nitrogen cycling, which only recently expanded to include the AOA. In extant ammonia oxidizers, bacterial and archaeal AMOs differ significantly, especially through structural disparities in transmembrane helices. Notably, recent work on putative additional archaeal subunits also suggests that although bacterial AMO complexes contain only three subunits (AmoABC), AOA may utilize six subunits (AmoABC and AmoXYZ [50]). The relationship between these structural distinctions and any functional differentiation is not yet understood, but commamox, other AOB and AOA do show wide-ranging substrate affinities for ammonia [9]. Within the AOA, the distribution of substrate affinities may be tied to ecological niche and phylogeny [7]. Diverging downstream biochemical pathways further compound the biochemical and ecological contrasts between AOA and AOB. Paired with processes like respiration and carbon fixation, which are carried out via different mechanisms in different groups, the relative contribution of AOA versus AOB affects models for nitrogen cycling and net primary productivity over time [4].

Whilst several steps in the nitrogen cycle (e.g. nitrogen fixation and denitrification) are performed by diverse organisms and are generally thought to have evolved early in Earth’s history (e.g. [51]), the limited set of lineages that perform ammonia oxidation may not have arisen until more recent time [4,48]. The phylogenetic divergence of AOA from closely related clades can help to untangle the relative timings of various evolutionary events. For example, of the traits highlighted in this paper, only aerobic respiration is present in the nearest relatives of extant AOA (so-far unnamed basal families of *Nitrososphaerales* known only from MAGs [33]), (Figs S2–S4). This suggests that the evolution of autotrophic ammonia oxidation in stem group AOA occurred relatively rapidly following the divergence of these groups. Whether aerobic respiration was independently acquired along this AOA stem branch several times or vertically inherited from the last common ancestor of all *Nitrososphaerales* has yet to be determined. Further investigation of this trait’s inheritance may be crucial for understanding the timing of the evolution of archaeal ammonia oxidation relative to the Great Oxygenation Event ~2.3 Gya when biologically meaningful concentrations of O_2_ first accumulated. In either case, the full diversity of characterized AOA appears to be adapted to relatively high O_2_ environments (given the utilization of a low-affinity A-family heme-copper oxidase [52] and the O_2_-tolerant 3HP/4HB carbon fixation pathway, e.g. [53]).

## Abbreviations

AMO: ammonia monooxygenase
AOA: ammonia-oxidizing archaea
AOB: ammonia-oxidizing bacteria
GTDB: Genome Taxonomy Database
MAGs: metagenome-assembled genomes
